# Neutrophils correlate with hypoxia microenvironment and promote progression of non-small-cell lung cancer

**DOI:** 10.1080/21655979.2021.1987820

**Published:** 2021-10-26

**Authors:** Chunyan Zhang, Bingxiang Tang, Jianping Hu, Xiang Fang, Hongzhi Bian, Junlei Han, Congxia Hou, Fang Sun

**Affiliations:** Respiratory and Critical Care Ward 1, Henan Provincial Chest Hospital, Zhengzhou, China

**Keywords:** Non-small-cell lung cancer, hypoxia, progression, gene signature, tumor-associated neutrophils, CXCL6

## Abstract

Hypoxia, a strong and selective pressure, has been involved in invasion, metastasis, and angiogenesis of tumor cells. Our study performed the transcriptome profiles of 666 non-small-cell lung cancer (NSCLC) patients. Various bioinformatic approaches were combined to evaluate the immune cell infiltration in the high hypoxia risk patients. In addition, in vitro experiments were performed to assess the effects of tumor-associated neutrophils (TANs) on NSCLC cells proliferation, migration and invasion and to reveal the underlying mechanisms. We divided NSCLC into two groups (Cluster1/2) based on the expression profiles of hypoxia-associated genes. Compared with the Cluster1 subgroup, the Cluster2 had a worse prognosis. Significant enrichment analysis revealed that PI3K/AKT/mTOR signaling pathway and TANs were highly related to hypoxia microenvironment. Eleven hypoxia-related genes (FBP1, NDST2, ADM, LDHA, DDIT4, EXT1, BCAN, IGFBP1, PDGFB, AKAP12, and CDKN3) were scored by LASSO COX regression to yield risk scores, and we revealed a significant difference in overall survival (OS) between the low- and high-risk groups. Mechanistically, CXCL6 in hypoxic cancer cells promoted the migration of TANs in vitro, and in turn promote NSCLC cells proliferation, migration and invasion. In summary, this study revealed a 11‐hypoxia gene signature that predicted OS of NSCLC patients, and improved our understanding of the role of TANs in hypoxia microenvironment.

## Introduction

Lung cancer is the leading cause of cancer death worldwide. Non-small cell lung cancer (NSCLC) comprises up to 80% of all lung cancer cases and has a poor prognosis [1]. Compared to the steadily increasing survival rates for most cancers, the survival rates for NSCLC are still unsatisfactory, mostly caused by resistance to anticancer treatment and metastasis [2]. Therefore, it is crucial to discover neo-markers to deliver greater prognostic value and understand the mechanisms of progression for NSCLC.

It has been demonstrated that tumor microenvironment (TME) plays a critical role in supporting the progression of NSCLC. The TME in NSCLC is composed of multiple components, including inflammatory cell infiltration, blood vessels, soluble factors, and hypoxic condition [3]. The hypoxic zone in solid tumors is invaded by a great many of immune-suppressant cells, such as tumor-associated neutrophils (TANs), myeloid derived suppressor cells (MDSCs), T-regulatory (Treg) cells and tumor-associated macrophages (TAMs) [4]. Emerging evidence suggests a strong link between the hypoxic TME and infiltrating immune cells. In hypoxic TME, lung cancer-derived exosomes reprogramed macrophages, which in turn promoted metastasis of lung cancer [5]. Hypoxia selectively upregulates PD-L1 on MDSCs via HIF-1α and blockade of PD-L1 abrogates MDSCs function [6]. As an important element of TME in NSCLC, TANs play crucial roles in supporting tumor growth. A pan-cancer study including NSCLC showed that TANs infiltration in the TME is a leading predictor of poor outcome [7]. Similarly, in NSCLC, a high ratio of neutrophils to lymphocytes is linked to a poor outcome [8]. However, the mechanisms involved in the association between TANs and hypoxic TME in the progression of cancer are still to a great extent unknown.

Hypoxia is a complex process that involves several hundred molecules [9]. Thus, models that integrate several hypoxia-related genes can improve the accuracy of prognostic predictions compared to a single gene. However, some of these characteristics were often uncertain and resulted from an unspecified genetic background. This study aimed to exploit machine learning methods to construct a robust prognostic gene panel. Based on the increased infiltration of TANs in high hypoxia risk tumors, we also investigated the specific mechanisms by which NSCLC cells recruit TANs under hypoxic conditions.

## Materials and methods

### Acquisition of lung cancer datasets

The set of sequence-based mRNA expression data (RNA-seq data) of NSCLC was downloaded from The Cancer Genome Atlas (TCGA) (https://cancergenome.nih.gov/). Another three gene expression arrays of human NSCLC datasets (GSE50081, GSE30219, and GSE37745) were downloaded from the Gene Expression Omnibus (GEO) (https://www.ncbi.nlm.nih.gov/geo). Moreover, clinicopathological information and survival data from four cohorts (TCGA, GSE50081, GSE30219, and GSE37745) were also obtained for further prognostic analysis.

### Identification of hypoxia status and assessing the immune infiltrates

To explore the function of hypoxia related genes in NSCLC, R package ‘ConsensusClusterPlus’ was applied to classified NSCLC patients into various clusters.

Infiltration levels of the abundances of immune cells in NSCLC were evaluated by CIBERSORT [10]. To avoid calculation errors, an immune infiltration estimation was performed using the ‘xCell’ R package [11].

### Gene set variation analysis (GSVA)

The actin pathway, MYC targets V1, IL-6/JAK2/STAT3 pathway, PI3K/Akt/MTOR signaling, hypoxia, epithelial-mesenchymal transition (EMT), and angiogenesis gene set files obtained from the ‘Molecular Signatures Database’ were utilized for GSVA using ‘GSVA’ packages for R [12].

### Establishment of prognostic model, receiver operating characteristic (ROC) curve and nomogram

A univariate Cox regression analysis was firstly performed on all of candidate genes to calculate the relationship between the expression of each gene and OS. The selected key genes were then further confirmed by the Lasso regression. The prognosis risk score was established, and a risk score based on the normalized expression data was generated. Patients were then divided into high- and low- risk groups according to the median cutoff of the prognosis risk score. The prognostic performance was evaluated by using time-dependent ROC curve analysis to estimate the predictive sensitivity and accuracy of our model. Using the survival and the rms package for R, age, gender, risk score, and stage were used to build a nomogram.

### Cell lines, cell culture and hypoxic exposure

The NSCLC cells (H-1299 and A549) were acquired from the ATCC. For normoxia experiments, the cells were cultured at 37°C and 5% CO2. For hypoxia experiments, the cells were cultured in a hypoxic chamber under hypoxic conditions (1% O2).

### Neutrophils from peripheral blood

Briefly, blood was obtained from NSCLC patients and erythrocytes were excised using the dextran sedimentation method. Using density centrifugation, neutrophils were clarified and re-suspended in RPMI 1640 containing 2% fetal bovine serum.

### Neutrophil from NSCLC tissues

Briefly, fresh NSCLC tissues were sliced into small pieces and digested in RPMI-1640 supplemented at 37°C for 30 min with 0.002% DNase I and 0.05% collagenase IV. The leukocytes were harvested and CD66b+ neutrophils were isolated using the EasySep PE Selection Kit (Stemcell Technologies) according to the manufacturer’s instructions.

### Cell proliferation and wound healing assay

To detect proliferation, 500 cells were plated on 6-well plates and maintained for 2 weeks. A 4% paraformaldehyde assay were used to stained with crystal violet.

Cells were loaded into 6-well plates for 48 h, and a wound was swabbed with a pipette tip. After incubation with serum-free medium for 48 h at 37°C, at the wound front migrating cells were pictured. The wound area was calculated with Image J software.

### Assessment of mRNA expression

Total RNA from NSCLC cells was extracted according to the manufacturer’s instructions by the Trizol Reagent. Subsequently, complementary DNA (cDNA) was obtained via reverse transcription for RT-PCR according to the SYBR-Green PCR Mix.

Primers sequences used in our study were as follows:

CXCL1 forward 5′-CCCAAGAACATCCAAAGTGTG-3′,

reverse 5′-CATTCTTGAGTGTGGCTATGAC-3′;

CXCL2 forward 5′-CTCAAGAACATCCAAAGTGTG-3′,

reverse 5′-ATTCTTGAGTGTGGCTATGAC-3′;

CXCL5 forward 5′-GGAAGGAAATTTGTCTTGATCC-3′,

reverse 5′-TTTCCTTGTTTCCACCGTC-3′;

CXCL6 forward 5′-CGTTACGCTGAGAGTAAACC-3′,

reverse 5′-GTTCTTCAGGGAGGCTACC-3′;

CXCL8 forward 5′-ACTCCAAACCTTTCCACCC-3′,

reverse 5′-CAATAATTTCTGTGTTGGCGC-3′;

CCL3 forward 5′-CGGTGTCATCTTCCTAACC-3′,

reverse 5′-TCGCTGACATATTTCTGGAC-3′.

### Plasmid constructs and transfections

For plasmid transfections, NSCLC cells were transfected with vectors of CXCR6-siRNA with the following target sequences: 5ʹ-ACGGATGTGTTCCTGGTGA −3ʹ or negative control RNA according to the manufacturer’s instructions using Lipofectamine 2000 (Roche, USA). Briefly, prior to treatment, Lipofectamine 2000/siRNA complexes were produced in reduced serum medium, OptiMEM (Invitrogen) at the recommended ratio of 20 pmol siRNA per 1ul Lipofectamine 2000. NSCLC cells were then treated with Lipofectamine 2000/siRNA complexes for 6 h before being replaced with RPMI 1640 containing 10% fetal bovine serum. NSCLC cells were also treated with Lipofectamine 2000/NC siRNA complexes as above descried.

### Enzyme-linked immunosorbent assay (ELISA)

According to the manufacturer’s instructions, the level of CXCL6 was measured using the Human CXCL6 ELISA Kit (Abnova). A curve of absorbance versus the concentration of CXCL6 in the standard wells was generated.

### Neutrophil chemotaxis assay

Neutrophil chemotaxis was assayed in a 3 μm Transwell system. Briefly, hypoxia or normoxia conditioned medium (CM) from NSCLC cells, CXCL6 in RPMI 1640 containing 2% FBS (Gibco) or hypoxia CM with CXCL6 neutralizing antibody was added to the lower wells. Neutrophils migrating into the lower chamber were gathered and numbered in the chambers.

### Transwell invasion assay

According to the manufacturer’s instructions, cells were placed in Boyden chambers coated with Matrigel diluted 1:8 with DMEM (Gibco) on the submembrane surface. After 48 hours, the aforementioned cells were immobilized in 4% paraformaldehyde and dyed with 0.5% crystal violet, and were evaluated under microscope by counting cells in five different areas per each condition.

## Statistical analysis

Intergroup comparisons were performed by one-way ANOVA or Student ‘s test. OS of the patients was determined by the Kaplan–Meier analysis and compared by the log-rank tests. The Cox proportional hazards regression model was used for univariate and multivariate survival analyses. LASSO analyses were applied to reduce the complexity of the model and select the significant hypoxia-related genes to construct the prognostic model. A value of *P* < 0.05 was regarded as statistically significant.

## Results

### Lung cancer patient characteristics and hypoxia-related gene identification

Three GEO databases with available OS data and clinical information (GSE50081, GSE30219, and GSE37745) were enrolled into one meta-cohort contained 666 lung cancer patients (Table S1). To classify the hypoxia status of lung cancer samples, we focused on 205 hypoxia-related genes that were obtained from previous research and Gene Set Enrichment Analysis (hallmark-hypoxia).

**Figure 1.**
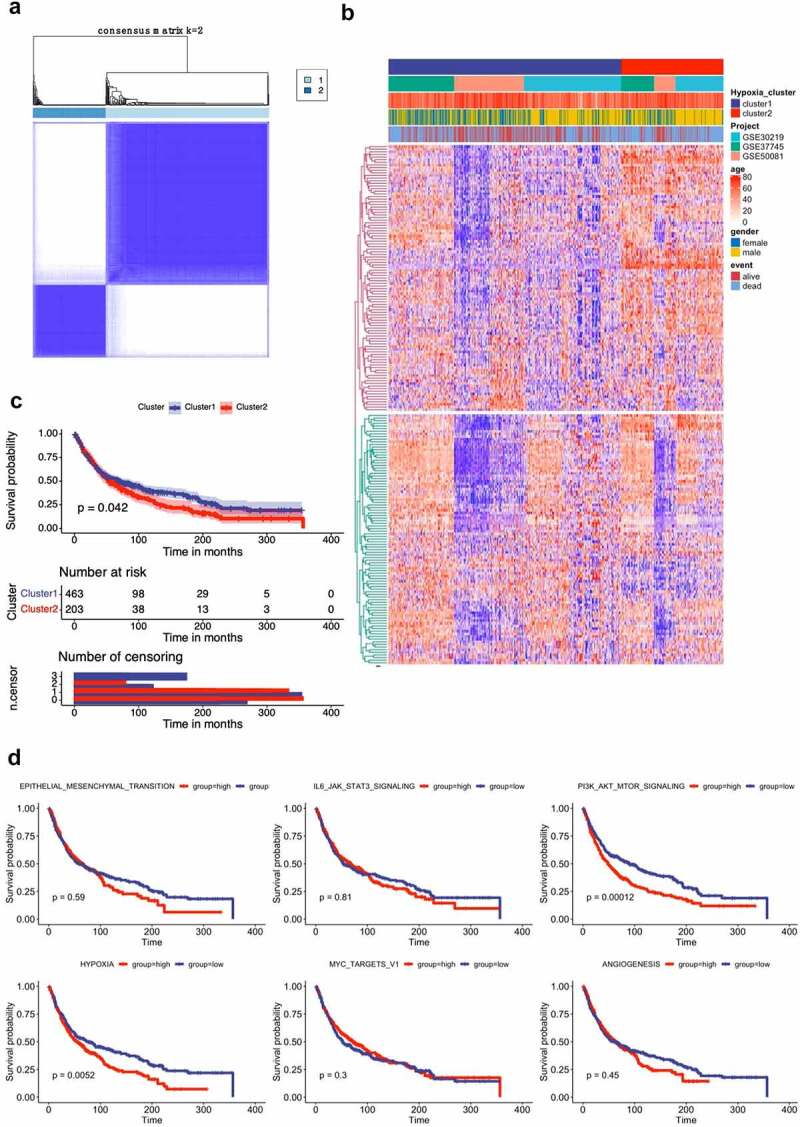
Lung cancer patient characteristics and hypoxia-related gene identification. (**A**) Consensus matrices of NSCLC patients for k=2 based on 205-hypoxia-related genes in three GEO cohort; (**B**) NSCLC cases are divided into two subtypes based on unsupervised analysis; (**C**) Differences in patient overall survival with two clusters; (**D**) Cox proportional hazard regression of survival months and survival status were performed using gene set enrichment scores for the six hypoxia-associated gene signatures. Log-rank test was used to determine significant p values

Then, we applied unsupervised clustering methods to classify 666 tumor patients into different molecular subgroups through 205 hypoxia related genes. Two distant subgroups, referred to as Cluster 1 and Cluster 2, were finalized (Figure 1a and 1B). The Kaplan-Meier curves showed significant differences in OS between the two clusters by log-rank test (Figure 1c). We further selected the GSE39582 datasets, which presents the most complete patient information, featuring the clinical discrepancies between these subgroups. Pathways involved in epithelial-mesenchymal transition (EMT), IL-6/JAK2/STAT3, PI3K/Akt/MTOR, Hypoxia, MYC targets V1 and Angiogenesis were activated in Cluster 2 (worse survival), whereas Cluster 1 (favorable survival), revealed enrichment of pathways correlated to tumor progression, including EMT, hypoxia, and angiogenesis (Figure. S1A). Further survival analysis showed that two gene sets (Hypoxia and PI3K/Akt/MTOR signaling) were associated worse survival (Figure 1d).

### The immune cell infiltration between high and low hypoxia risk NSCLC patients

**Figure 2.**
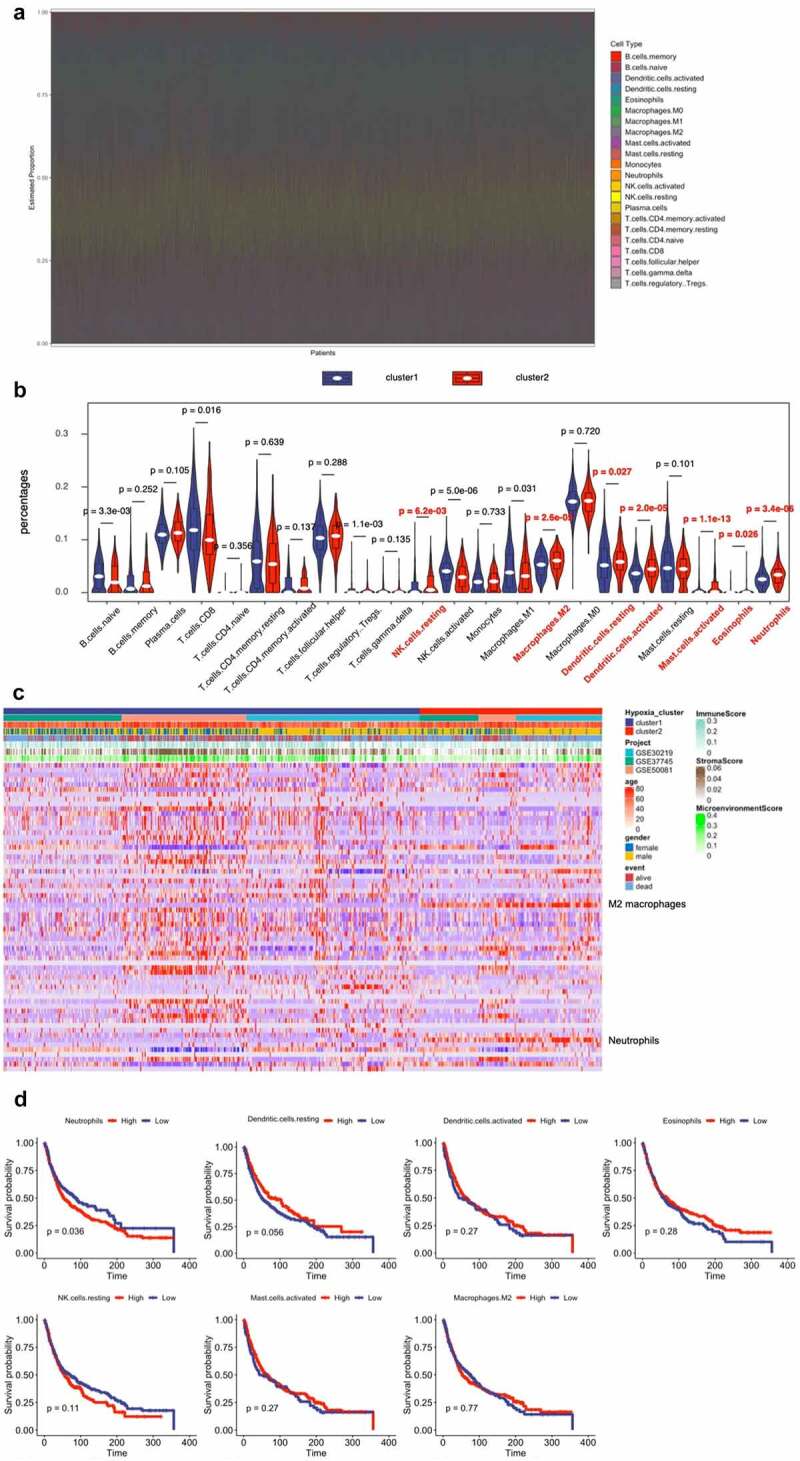
Immune Landscape Between Low and High Hypoxia Risk NSCLC Patients. (**A**) and (**B**) The 22 immune cells proportions obtained from 666 lung cancer patients in GEO databases; (**C**) Rows of the heatmap show expression of TME-infiltrating cell signatures calculated by xCell; (**D**) Kaplan-Meier plots of infiltrating immune cells with differential expression as described above

Growing evidence shown that the hypoxic microenvironment may protect tumors from anti-tumor immune responses by promoting immune escape and suppressing anti-tumor immune cells. Using the CIBERSORT method, we assessed differences of 22 immune cell infiltration between Cluster 1 and Cluster 2 patients. Figure 2a and S2A summarizes the results obtained from 666 lung cancer patients in GEO databases. The proportion of immunosuppressive cells was significantly higher in Cluster 2 with high hypoxia risk (e.g., macrophages M2 and neutrophils), and NK cells resting (Figures 2B). Although Tregs cells did not differ between high and low hypoxic risk tumors, inactivated NK cells and immunosuppressive cells may drive the immunosuppressive microenvironment. Transcriptome file of three GEO databases was applied on the ESTIMATE method to estimate the infiltration of different immune cells. Cluster 2 with high hypoxia risk was significantly enriched in immunosuppressive immune cells, such as macrophages M2 and neutrophils as mentioned above (Figure 2c). We determined if any of the above immune cells with differential expression correlated with patient survival. This analysis showed that only neutrophils were associated with worse survival (Figure 2d). Therefore, targeting hypoxia may have significant clinical implications in improving immunotherapy.

### Validation of hypoxia-related gene set in the TCGA cohort

**Figure 3.**
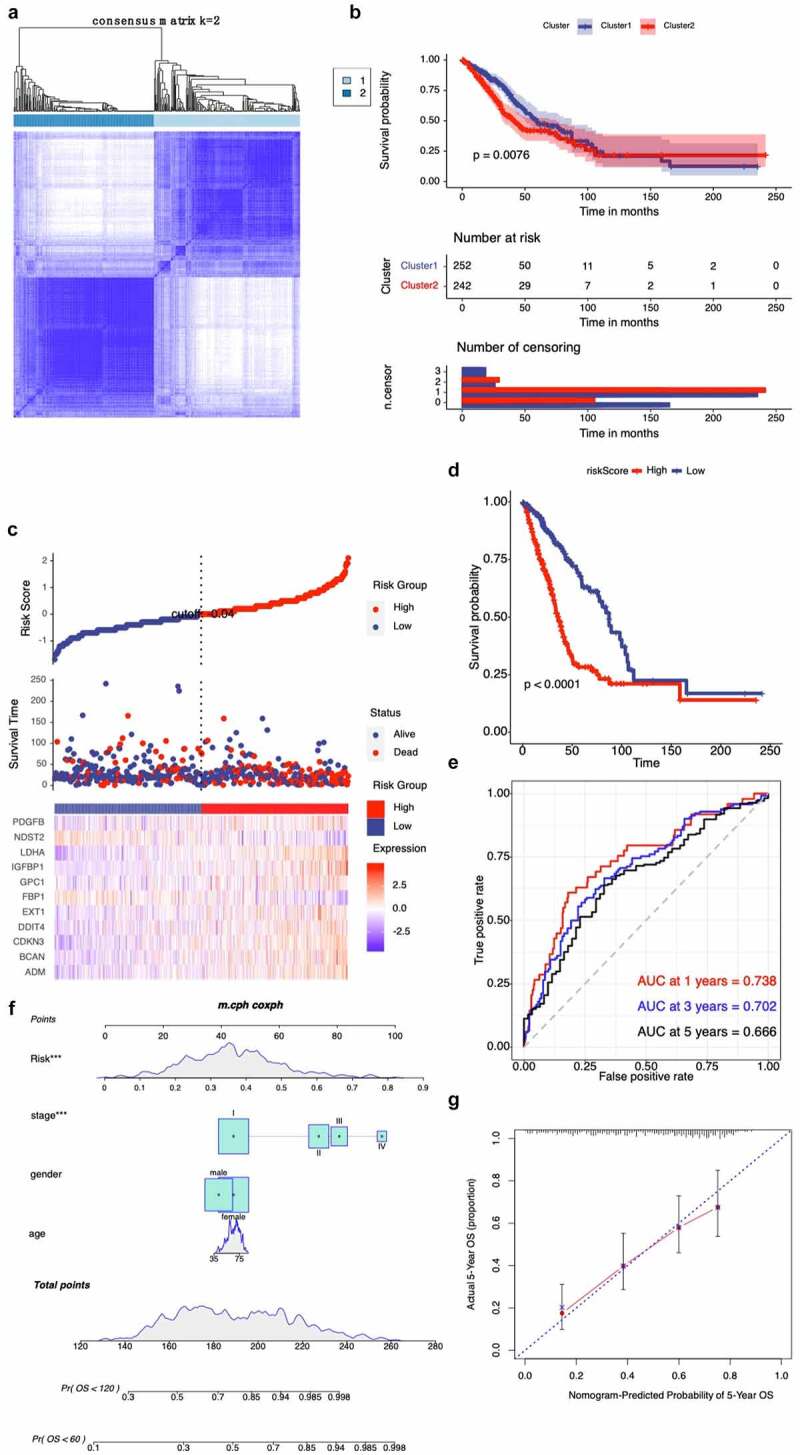
Validation of hypoxia-related gene set in the TCGA cohort. (**A**) Consensus matrices of NSCLC patients for k=2 based on 205-hypoxia-related genes in the TCGA cohort; (**B**) Differences in patient overall survival with two clusters; (**C**) Establishment of a prognosis-predictive model dividing patients into high and low risk groups; (**D**) Differences in patient overall survival with high and low risk groups; (**E**) Time-dependent ROC curves for the risk score in the TCGA dataset for predicting 1, 3, and 5-year OS; (**F**) Nomogram based on risk score, age, gender, and stage; (**G**) Calibration plots of the nomogram for predicting the probability of OS at 5 years in the TCGA dataset

The hypoxia-related gene set was further validated by the TCGA cohort, and we condensed into two distinct hypoxia-related subtypes by applying consensus clustering analysis (Figure 3a). Patients in Cluster1 had a higher OS, while patients in Cluster2 yielded a poorer prognosis outcome (Figure 3b). All these genes were subjected to Cox regression analysis. A total of 60 genes were markedly correlated with the OS of TCGA, and LASSO Cox regression was used to filter out less relevant prognostic genes (Fig. S2B). The optimal gene signature consisting of 11 prognostic genes were identified (Fig. S2C). Using the corresponding coefficients of 11 prognostic genes, the individual-level risk score formula for each patient was calculated as following: risk score = – 0.24× FBP1 – 0.90× NDST2 + 0.13× ADM + 1.02× LDHA + 0.05× DDIT4 + 0.82× EXT1 + 0.06× BCAN + 0.12× IGFBP1 + 0.20× PDGFB + 0.22× AKAP12 + 0.26× CDKN3. As shown in Figure 3c, the heatmap revealed that the expression level of 11 genes (FBP1, NDST2, ADM, LDHA, DDIT4, EXT1, BCAN, IGFBP1, PDGFB, AKAP12, and CDKN3) varied accompanying with the higher risk scores. In addition, we calculated the relationship between risk score and cancer-related mortality, and the patients who had higher risk scores were supposed to have a shorter OS than lower risk group, suggesting that these 11 genes might be considered as risk genes in NSCLC (Figure 3d). Time-dependent ROC curve showed that our 11 prognostic gene model exhibited good performance and accuracy in prognosis prediction either for 1-year (AUC = 0.738), 3-year (AUC = 0.702) and 5-year (AUC = 0.666) OS of NSCLC patients (Figure 3e). We generated a nomogram with a concordance index of 0.733 to predict the probability of 5 and 10-year OS, by incorporating the 11-hypoxia gene signature, age, gender, and TNM stage (figure 3f). The calibration curve showed that the nomogram performed a high level of accuracy (Figure 3g).

### CXCL6 is the key chemokine for the recruitment of TANs in hypoxic NSCLC cells

**Figure 4.**
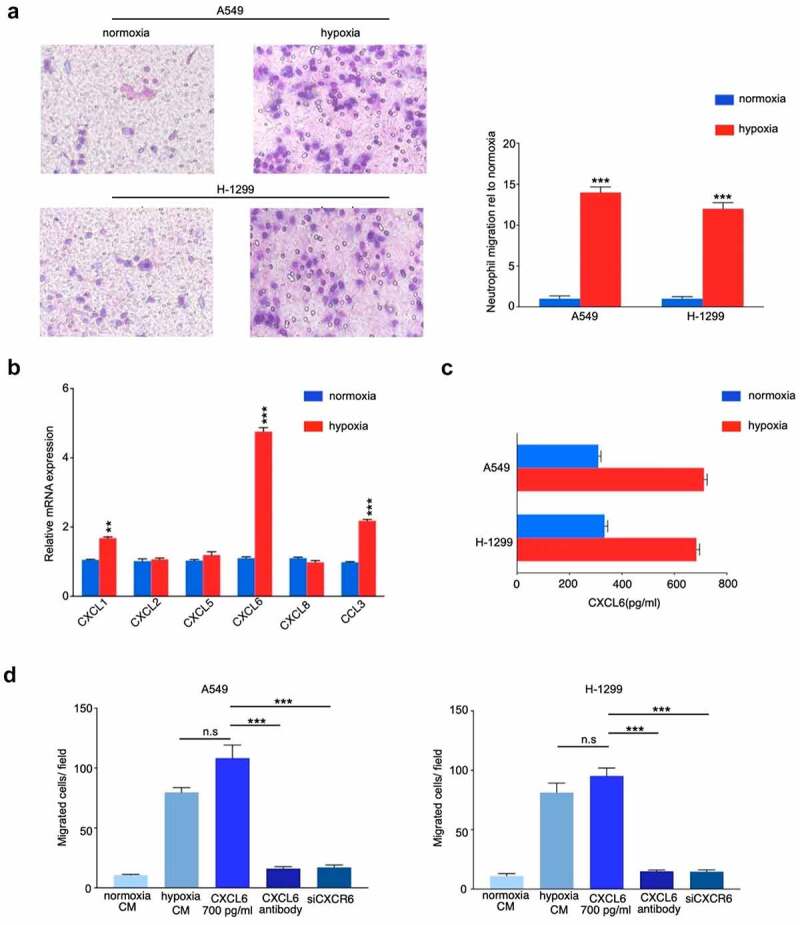
CXCL6 is the critical chemokine induced by hypoxic NSCLC cell to recruit TANs derived from NSCLC tissues. (**A**) Quantification of neutrophil migration as assessed by transwell assays; (**B**) and (**C**) Expression of CXCL6 in hypoxic or normoxic NSCLC cells was examined by real-time PCR and ELISA; (**D**) Quantification of neutrophil migration as assessed by transwell assays

Based on the aforementioned important role played by TANs infiltration in hypoxic TME, we further examined the effect of conditioned media on neutrophils in hypoxia-treated NSCLC cells. As shown in Figure 4a, CM from hypoxia-induced A549 and H-1299 cells recruited more TANs derived from NSCLC tissues than CM from normoxic cells. To further screen the possible chemokines, candidate chemokines were identified through RT-qPCR. Hypoxia facilitated NSCLC cells to generate more neutrophil chemokines, and CXCL6 increased the most among all the increased chemokines (CXCL1, CXCL2, CXCL5, CXCL6, CXCL8, and CCL3) as detected by RT-PCR (Figure 4b). Consistently, ELISA revealed that the expression of CXCL6 was significantly increased under hypoxia condition (Figure 4c). Transwell migration assays were performed to determine that migration of TANs enhanced by hypoxic NSCLC cells was almost identical to that of 700 pg/ml CXCL6 (Figure 4d). In agreement, migration of TANs of hypoxic NSCLC was repressed by a CXCL6 antibody. Furthermore, obstruction of the CXCL6 receptor CXCR6 (siCXCR6) in TANs markedly compromised the migration of TANs. Similar results were obtained by the exploration of TANs derived from peripheral blood (Figure. S3A-D).

### TANs promote NSCLC cells proliferation, migration and invasion

**Figure 5.**
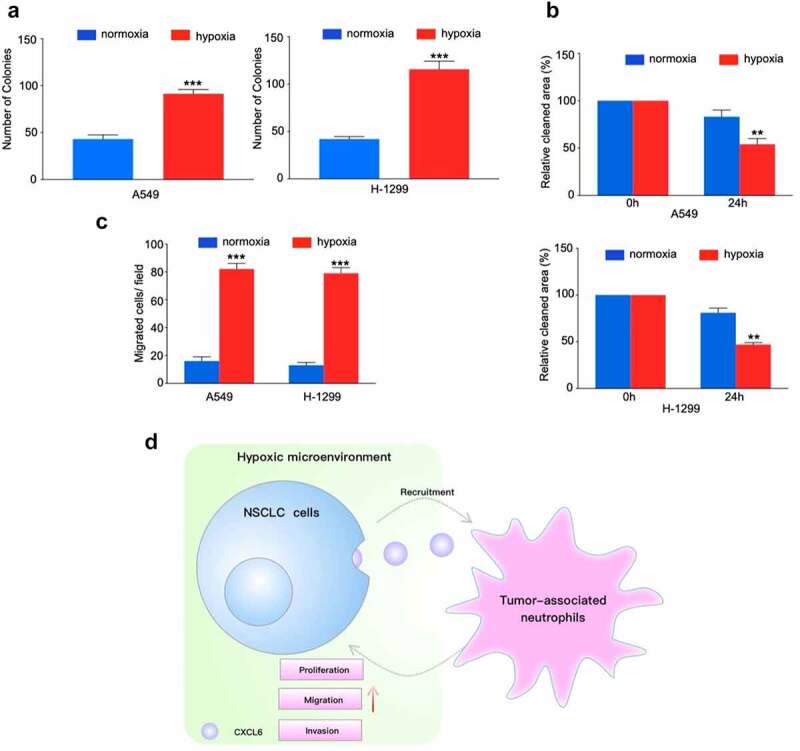
TANs derived from NSCLC tissues promote NSCLC cells proliferation, migration and invasion. (**A**), (**B**) and (**C**): NSCLC cells cocultured with TANs or alone were subjected to colony formation, wound healing, and transwell invasion assays; (**D**) Schematic illustration of the crosstalk between CXCL6-overexpressing NSCLC cells and TANs in the TME

To examine the effect of neutrophils on NSCLC cells biology, we cocultured NSCLC cell lines with TANs derived from NSCLC tissues. Proliferation analysis demonstrated that TANs promoted NSCLC cells growth in vitro (Figure 5a). Moreover, compared with control, TANs significantly enhanced the migration and invasion abilities of A549 and H-1299 cells (Figure 5b and c). Similar results were obtained by the exploration of TANs derived from peripheral blood (Figure. S4A-C).

In this study, we verified hypoxia upregulated neutrophils recruitment and constructed a novel hypoxia associated gene signature that could improve the individualized prognosis prediction in NSCLC. Moreover, hypoxia increased CXCL16 production and attracting TANs. CXCL6-mediated TANs infiltration contributes to hypoxic-related cancer progression (Figure 5d).

## DISCUSSION

Given the wide variation in prognostic outcomes of NSCLC, it is of great importance to build a robust classifier to segment patients with different risks and prognosis, which is essential to maximize the benefits of accurate assessment, individual therapy and timely long-term follow-up. Numerous data indicate that hypoxia and TME are involved in processes that promote the development of tumor cells and a more malignant phenotype [4,13]. Consistent with criteria prognostic parameters like nodal status, tumor stage and tumor grade, hypoxia and immune cells infiltrating TME have been proposed as prognostic factors for patient outcome [14,15]. Although methods such as PET imaging and immunohistochemical marker expression assays have been initially implemented to detect the degree of hypoxia in patients’ tumors, the specific identification methods are still fraught with many unknowns. In this study, the integrated mining of transcriptional profiles and microenvironment features was intended to establish a tool to solve this vital clinical issue.

In this study, we profiled the mRNA expression of 205 hypoxia-associated genes in three GEO databases. Two NSCLC subgroups Cluster 1/2 were identified by consistent clustering on the basis of hypoxia-related genes. The Cluster 1/2 subgroup not only affects disease prognosis and key signaling pathways, but it influences immune cells infiltration. Bioinformatic approach uncovered that hypoxia condition mainly associated with the PI3K/Akt/MTOR signaling pathway, the JAK2/STAT3 signaling pathway, the MYC targets V1 signaling pathway, and angiogenesis. Interestingly, a number of studies have found that hypoxia plays a role in the induction of tumor cell growth and metastasis by PI3K/Akt signaling and MYC target V1 signaling [16–18]. There is also much evidence to support the interaction between hypoxia and angiogenesis [19–21]. Moreover, we used Cox and LASSO regression to develop an 11-gene prognostic signature for TCGA cohort. The risk scores were derived by integrating mRNA expression levels and risk coefficients for 11 hypoxia-related genes, and significantly classified prognosis in NSCLC patients into low and high-risk clusters. There are a number of clinically available multigene based risk models that can predict the prognosis of NSCLC. Zhu and colleagues recently reported a 22-autophagy-related signature based on overall survival in patients with lung adenocarcinoma [22]. Recently, Wu et al. reported a seven long non‐coding RNAs prognostic model to predict OS in NSCLC patients. So far, there were limited prognostic models based on hypoxia-associated genes for patients with NSCLC. In an effort to exploit hypoxia-induced epithelial-mesenchymal transition gene signatures associated with clinical outcomes in NSCLC, Gao et al. demonstrated a 17 gene prognostic panel for NSCLC [23]. Our model shows good accuracy and stability in clinical outcome prediction either for 1-year (AUC = 0.738), 3-year (AUC = 0.702) and 5-year (AUC = 0.666) OS of NSCLC patients. Our data also revealed that all 11 genes in our model were associated with poor prognosis in NSCLC patients. These discovered hypoxia-associated genes have been repeatedly reported in cancer [24–27]. Previous studies reported that hypoxia-induced GBE1 expression promotes tumor progression by repressing FBP1 activities in lung adenocarcinoma [28]. In contrast, in the present study, we found a positive coefficient for FBP1, indicating that FBP1 was considered as risk gene in NSCLC. The underlying molecular mechanisms deserve further investigations.

Accumulating evidence indicates that hypoxia is an important feature of TME and that it can drive progression and metastasis by facilitating suppressive cells (TANs, Tregs, and TAMs), and producing immunosuppressive molecules. CIBERSORT revealed that patients in Cluster 2 with high hypoxia risk had significantly more abundant infiltrative TANs and M2 macrophages phenotype. Furthermore, TANs were associated with a poor prognosis, suggesting that our hypoxia model may predict the immune microenvironment. Hypoxia not only tightly regulates the production of specific chemokines, it also controls their action by regulating their receptors [29,30]. Chemokines play an important role in regulating tumor immunity [31]. Our findings therefore provide insight into the underlying mechanisms of recruitment-related chemokines in TANs infiltration, and found that the production of CXCL6 increased in NSCLC cells under hypoxic condition, and the blockade of CXCL6 almost halted TANs migration, suggesting that the recruitment of TANs is mediated mainly by CXCL6. CXCL6 induced proliferation and metastasis of lung cancer cell lines was confirmed in other studies, and the role of CXCL6 in the TME of NSCLC is unclear [32,33]. Our study broadened the understanding of CXCL6 in NSCLC progression. As previously mentioned, multiple studies have noted that TANs appear to mostly develop a pro-tumorigenic phenotype [34,35]. In this study, we revealed TANs enhanced the proliferative and invasive ability of NSCLC cells in vitro. Experimental studies are needed to further clarify the molecular mechanisms underlying the TANs related pro-tumorigenic phenotype in NSCLC.

### Conclusions

In conclusion, we identified a hypoxia model based on 11-hypoxia gene signature, and investigated the association between hypoxic condition and infiltrating immune cells, especially TANs in NSCLC patients. Then, we confirmed that CXCL6 is a strong chemotactic cytokine for TANs infiltration in NSCLC and observed that the role of TANs on NSCLC proliferation, migration and invasion. Thus, our study provided more comprehensive insight into how hypoxia status influence on prognosis and the TME, and may help clinicians develop individualized hypoxia-targeted therapies in NSCLC.

## Supplementary Material

Supplemental Material

## Data Availability

The data that support the findings of this study are openly available in Gene Expression Omnibus (http://www.ncbi. nlm.nih.gov/geo) and University of California Santa Cruz Xena browser (UCSC Xena: http://xena.ucsc.edu/).

## Abbreviations

NSCLC: non-small-cell lung cancer; TANs: tumor-associated neutrophils; OS: overall survival; ROC: receiver operating characteristic; GSVA: Gene set variation analysis; GEO: Gene Expression Omnibus; TME: tumor microenvironment; TCGA: The Cancer Genome Atlas

## References

[1] Siegel RL, Miller KD, Jemal A. Cancer statistics, 2018. CA Cancer J Clin. 2018;68:7–30. 29313949 10.3322/caac.21442

[2] Peters S, Gettinger S, Johnson ML, et al. Phase II trial of Atezolizumab as first-line or subsequent therapy for patients with programmed death-ligand 1-selected advanced non-small-cell lung cancer (BIRCH). J Clin Oncol. 2017;35:2781–2789. 28609226 10.1200/JCO.2016.71.9476 PMC5562171

[3] Lambrechts D, Wauters E, Boeckx B, et al. Phenotype molding of stromal cells in the lung tumor microenvironment. Nat Med. 2018;24:1277–1289. 29988129 10.1038/s41591-018-0096-5

[4] Jing X, Yang F, Shao C, et al. Role of hypoxia in cancer therapy by regulating the tumor microenvironment. Mol Cancer. 2019;18:157. 31711497 10.1186/s12943-019-1089-9 PMC6844052

[5] Hsu YL, Hung JY, Chang WA, et al. Hypoxic lung-cancer-derived extracellular vesicle microRNA-103a increases the oncogenic effects of macrophages by targeting PTEN. Mol Ther. 2018;26:568–581. 29292163 10.1016/j.ymthe.2017.11.016 PMC5835028

[6] Noman MZ, Desantis G, Janji B, et al. PD-L1 is a novel direct target of HIF-1α, and its blockade under hypoxia enhanced MDSC-mediated T cell activation. J Exp Med. 2014;211:781–790. 24778419 10.1084/jem.20131916 PMC4010891

[7] Gentles AJ, Newman AM, Liu CL, et al. The prognostic landscape of genes and infiltrating immune cells across human cancers. Nat Med. 2015;21:938–945. 26193342 10.1038/nm.3909 PMC4852857

[8] Templeton AJ, McNamara MG, Šeruga B, et al. Prognostic role of neutrophil-to-lymphocyte ratio in solid tumors: a systematic review and meta-analysis. J Natl Cancer Inst. 2014;106:dju124. 24875653 10.1093/jnci/dju124

[9] Lin W, Wu S, Chen X, et al. Characterization of hypoxia signature to evaluate the tumor immune microenvironment and predict prognosis in glioma groups. Front Oncol. 2020;10:796. 32500034 10.3389/fonc.2020.00796 PMC7243125

[10] Newman AM, Liu CL, Green MR, et al. Robust enumeration of cell subsets from tissue expression profiles. Nat Methods. 2015;12:453–457. 25822800 10.1038/nmeth.3337 PMC4739640

[11] Aran D, Hu Z. Butte AJJGb: xCell: digitally portraying the tissue cellular heterogeneity landscape. Genome biology. 2017;18:1–14. 10.1186/s13059-017-1349-1 PMC5688663 29141660

[12] Hänzelmann S, Castelo R, Guinney J. GSVA: gene set variation analysis for microarray and RNA-seq data. BMC Bioinformatics. 2013;14:7. 23323831 10.1186/1471-2105-14-7 PMC3618321

[13] DeBerardinis RJ, Phimister EG. Tumor microenvironment, metabolism, and immunotherapy. N Engl J Med. 2020;382:869–871. 32101671 10.1056/NEJMcibr1914890

[14] Jubb AM, Buffa FM, Harris AL. Assessment of tumour hypoxia for prediction of response to therapy and cancer prognosis. J Cell Mol Med. 2010;14:18–29. 19840191 10.1111/j.1582-4934.2009.00944.x PMC3837600

[15] Bao X, Shi R, Zhao T, et al. Mast cell-based molecular subtypes and signature associated with clinical outcome in early-stage lung adenocarcinoma. Mol Oncol. 2020;14:917–932. 32175651 10.1002/1878-0261.12670 PMC7191192

[16] Dou C, Zhou Z, Xu Q, et al. Hypoxia-induced TUFT1 promotes the growth and metastasis of hepatocellular carcinoma by activating the Ca(2+)/PI3K/AKT pathway. Oncogene. 2019;38:1239–1255. 30250300 10.1038/s41388-018-0505-8

[17] Xue G, Yan HL, Zhang Y, et al. c-Myc-mediated repression of miR-15-16 in hypoxia is induced by increased HIF-2α and promotes tumor angiogenesis and metastasis by upregulating FGF2. Oncogene. 2015;34:1393–1406. 24704828 10.1038/onc.2014.82

[18] Li Z, Rich JN. Hypoxia and hypoxia inducible factors in cancer stem cell maintenance. Curr Top Microbiol Immunol. 2010;345:21–30. 20582533 10.1007/82_2010_75

[19] Patra K, Jana S, Sarkar A, et al. The inhibition of hypoxia-induced angiogenesis and metastasis by cinnamaldehyde is mediated by decreasing HIF-1α protein synthesis via PI3K/Akt pathway. Biofactors. 2019;45:401–415. 30854715 10.1002/biof.1499

[20] Kaur B, Khwaja FW, Severson EA, et al. Hypoxia and the hypoxia-inducible-factor pathway in glioma growth and angiogenesis. Neuro Oncol. 2005;7:134–153. 15831232 10.1215/S1152851704001115 PMC1871894

[21] Goudar RK, Vlahovic G. Hypoxia, angiogenesis, and lung cancer. Curr Oncol Rep. 2008;10:277–282. 18778551 10.1007/s11912-008-0043-6

[22] Liu Y, Wu L, Ao H, et al. Prognostic implications of autophagy-associated gene signatures in non-small cell lung cancer. Aging (Albany NY). 2019;11:11440–11462. 31811814 10.18632/aging.102544 PMC6932887

[23] Chen YL, Zhang Y, Wang J, et al. A 17 gene panel for non-small-cell lung cancer prognosis identified through integrative epigenomic-transcriptomic analyses of hypoxia-induced epithelial-mesenchymal transition. Mol Oncol. 2019;13:1490–1502. 30973670 10.1002/1878-0261.12491 PMC6599842

[24] Du F, Sun L, Chu Y, et al. DDIT4 promotes gastric cancer proliferation and tumorigenesis through the p53 and MAPK pathways. Cancer Commun (Lond). 2018;38:45. 29976242 10.1186/s40880-018-0315-y PMC6034313

[25] He P, Li K, Li SB, et al. Upregulation of AKAP12 with HDAC3 depletion suppresses the progression and migration of colorectal cancer. Int J Oncol. 2018;52:1305–1316. 29484387 10.3892/ijo.2018.4284

[26] Liu GM, Li Q, Zhang PF, et al. Restoration of FBP1 suppressed Snail-induced epithelial to mesenchymal transition in hepatocellular carcinoma. Cell Death Dis. 2018;9:1132. 30429463 10.1038/s41419-018-1165-x PMC6235921

[27] Zheng F, Tang Q, Zheng XH, et al. Inactivation of Stat3 and crosstalk of miRNA155-5p and FOXO3a contribute to the induction of IGFBP1 expression by beta-elemene in human lung cancer. Exp Mol Med. 2018;50:121. 10.1038/s12276-018-0146-6 PMC6135838 30209296

[28] Li L, Yang L, Fan Z, et al. Hypoxia-induced GBE1 expression promotes tumor progression through metabolic reprogramming in lung adenocarcinoma. Signal Transduct Target Ther. 2020;5:54. 32439898 10.1038/s41392-020-0152-8 PMC7242448

[29] Facciabene A, Peng X, Hagemann IS, et al. Tumour hypoxia promotes tolerance and angiogenesis via CCL28 and T(reg) cells. Nature. 2011;475:226–230. 21753853 10.1038/nature10169

[30] Chen XJ, Deng YR, Wang ZC, et al. Hypoxia-induced ZEB1 promotes cervical cancer progression via CCL8-dependent tumour-associated macrophage recruitment. Cell Death Dis. 2019;10:508. 31263103 10.1038/s41419-019-1748-1 PMC6602971

[31] Nagarsheth N, Wicha MS, Zou W. Chemokines in the cancer microenvironment and their relevance in cancer immunotherapy. Nat Rev Immunol. 2017;17:559–572. 28555670 10.1038/nri.2017.49 PMC5731833

[32] Li J, Tang Z, Wang H, et al. CXCL6 promotes non-small cell lung cancer cell survival and metastasis via down-regulation of miR-515-5p. Biomed Pharmacother. 2018;97:1182–1188. 29136957 10.1016/j.biopha.2017.11.004

[33] Zhu YM, Bagstaff SM, Woll PJ. Production and upregulation of granulocyte chemotactic protein-2/CXCL6 by IL-1beta and hypoxia in small cell lung cancer. Br J Cancer. 2006;94:1936–1941. 16721367 10.1038/sj.bjc.6603177 PMC2361351

[34] Fridlender ZG, Sun J, Kim S, et al. Polarization of tumor-associated neutrophil phenotype by TGF-beta: “N1” versus “N2” TAN. Cancer Cell. 2009;16:183–194. 19732719 10.1016/j.ccr.2009.06.017 PMC2754404

[35] Andzinski L, Kasnitz N, Stahnke S, et al. Type I IFNs induce anti-tumor polarization of tumor associated neutrophils in mice and human. Int J Cancer. 2016;138:1982–1993. 26619320 10.1002/ijc.29945

