# Supplementary material for: Neutrophils correlate with hypoxia microenvironment and promote progression of non-small-cell lung cancer
Source: Bioengineered. 2021 Oct 26;12(1):8872–84. doi: 10.1080/21655979.2021.1987820 (PMC8806964; doi:10.1080/21655979.2021.1987820)
Supplement: Supplemental Material [file KBIE_A_1987820_SM5135.docx]

**
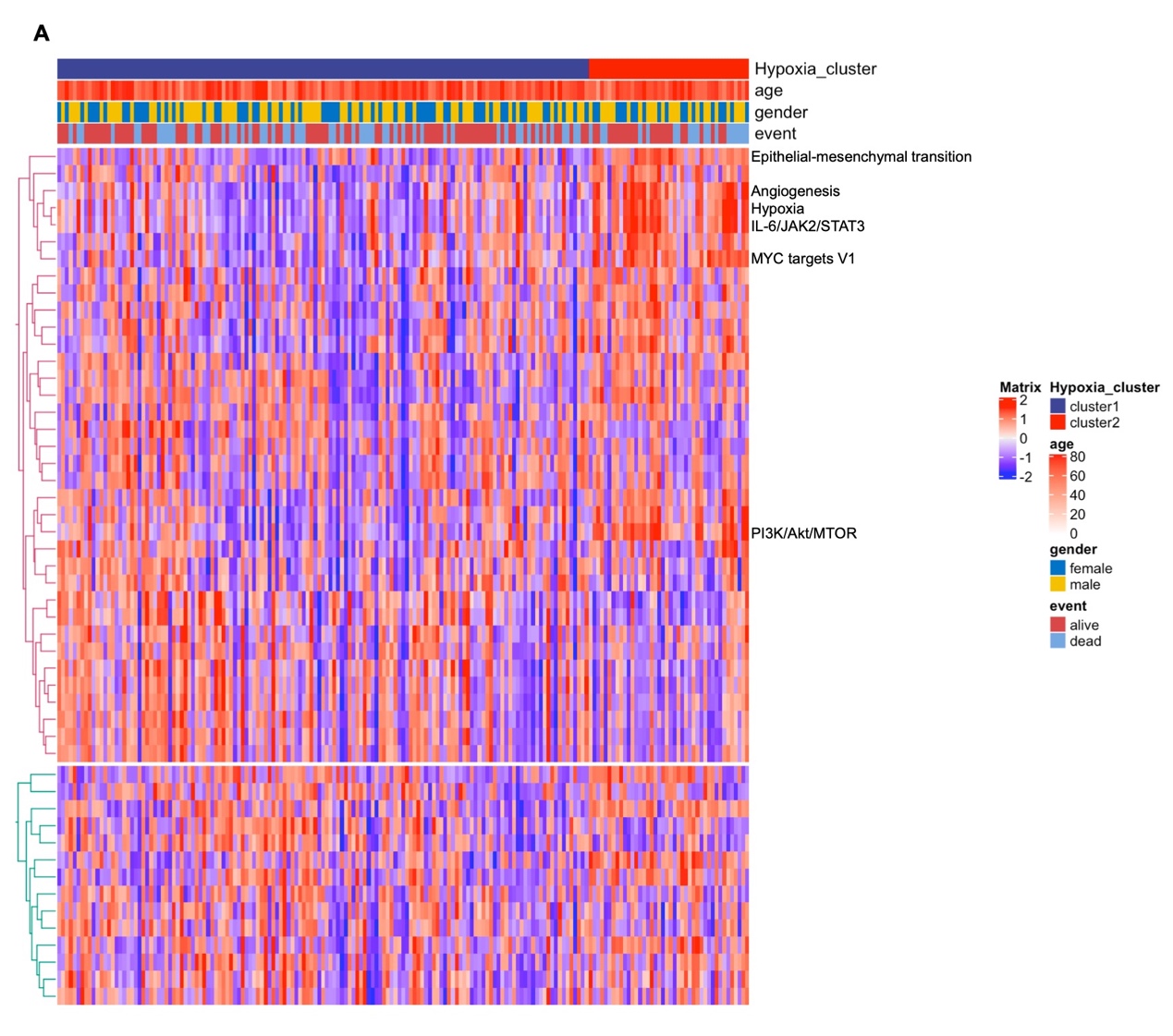
**

**FIGURE S1**

**
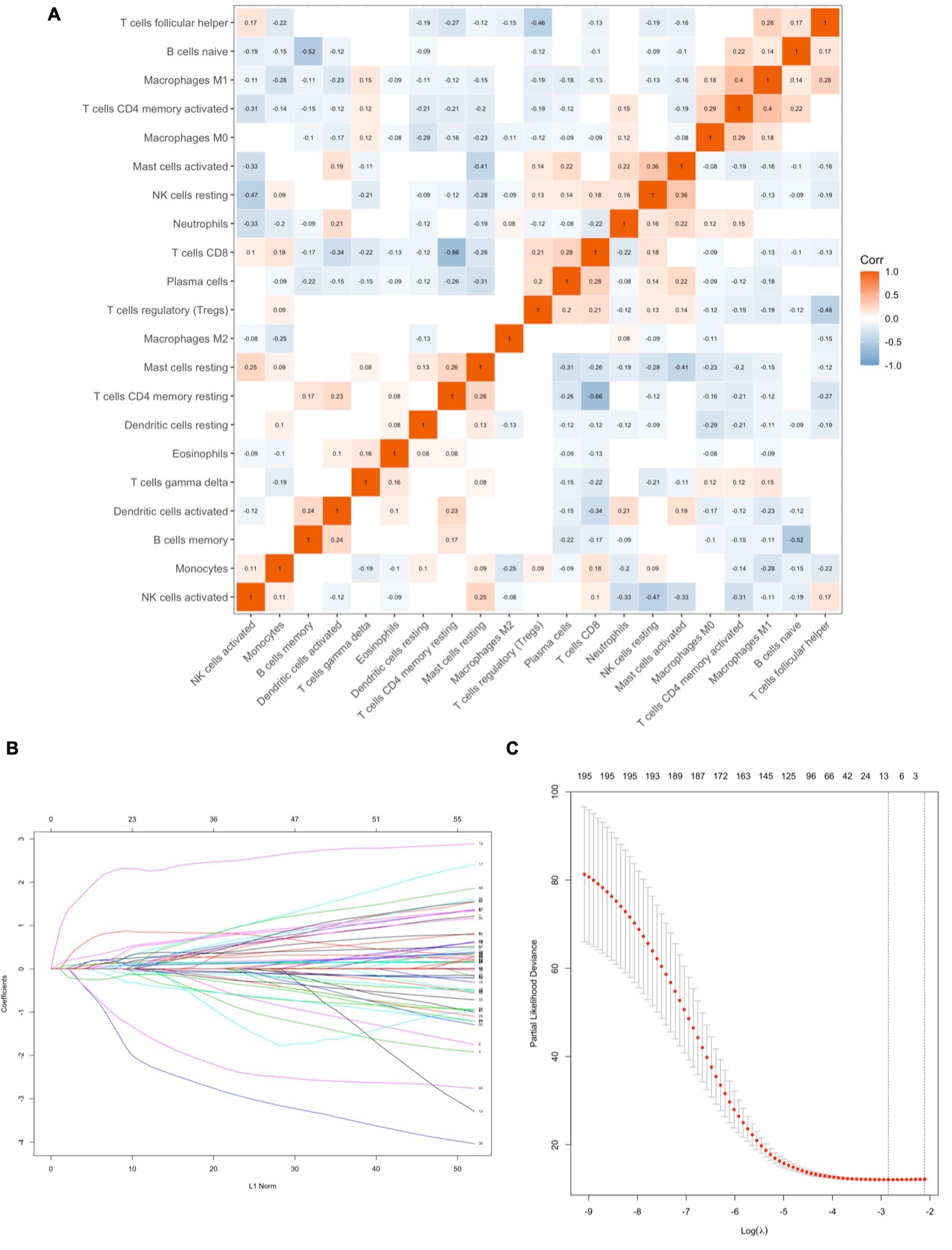
**

**FIGURE S2**

**
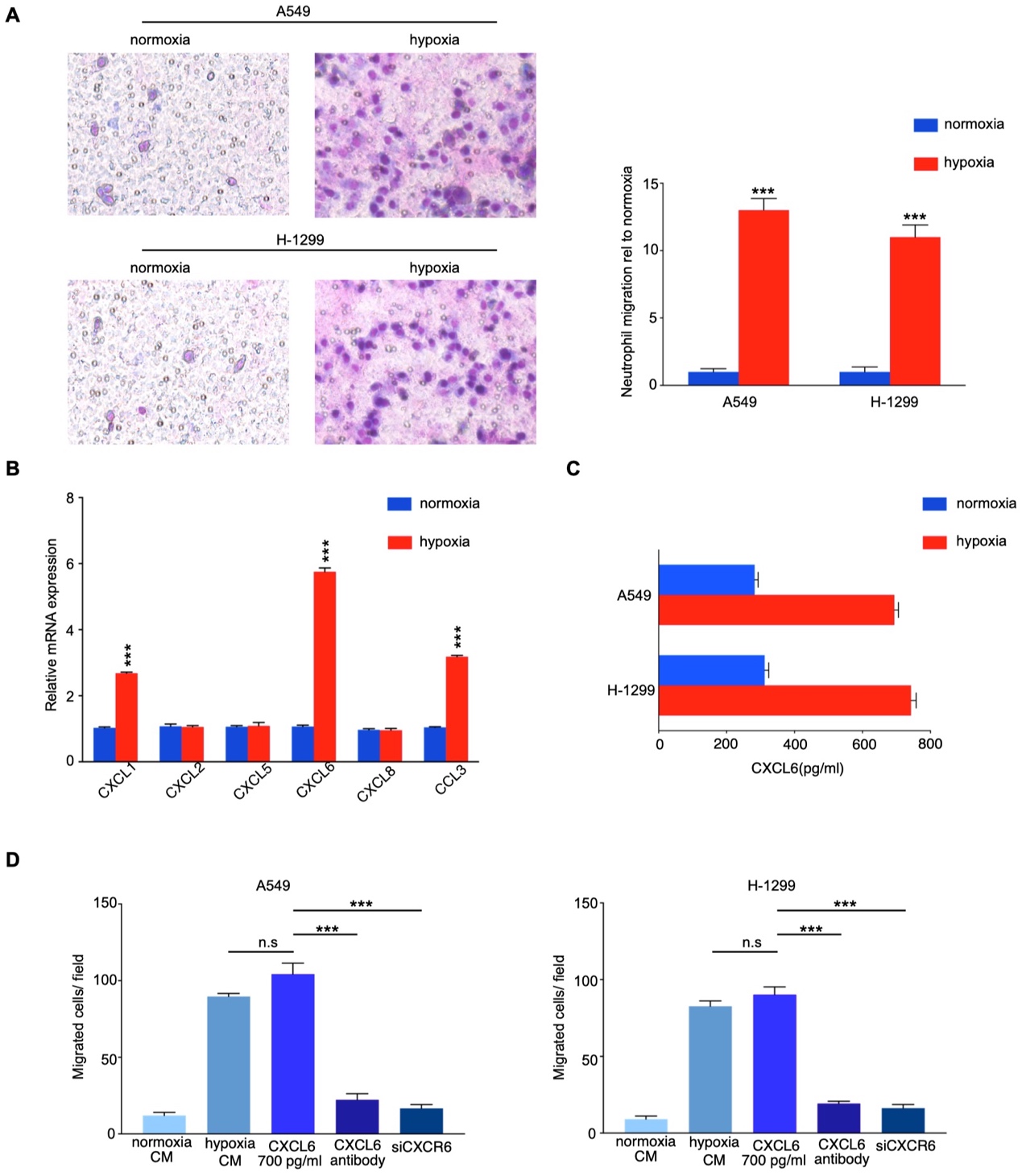
**

**FIGURE S3**

**
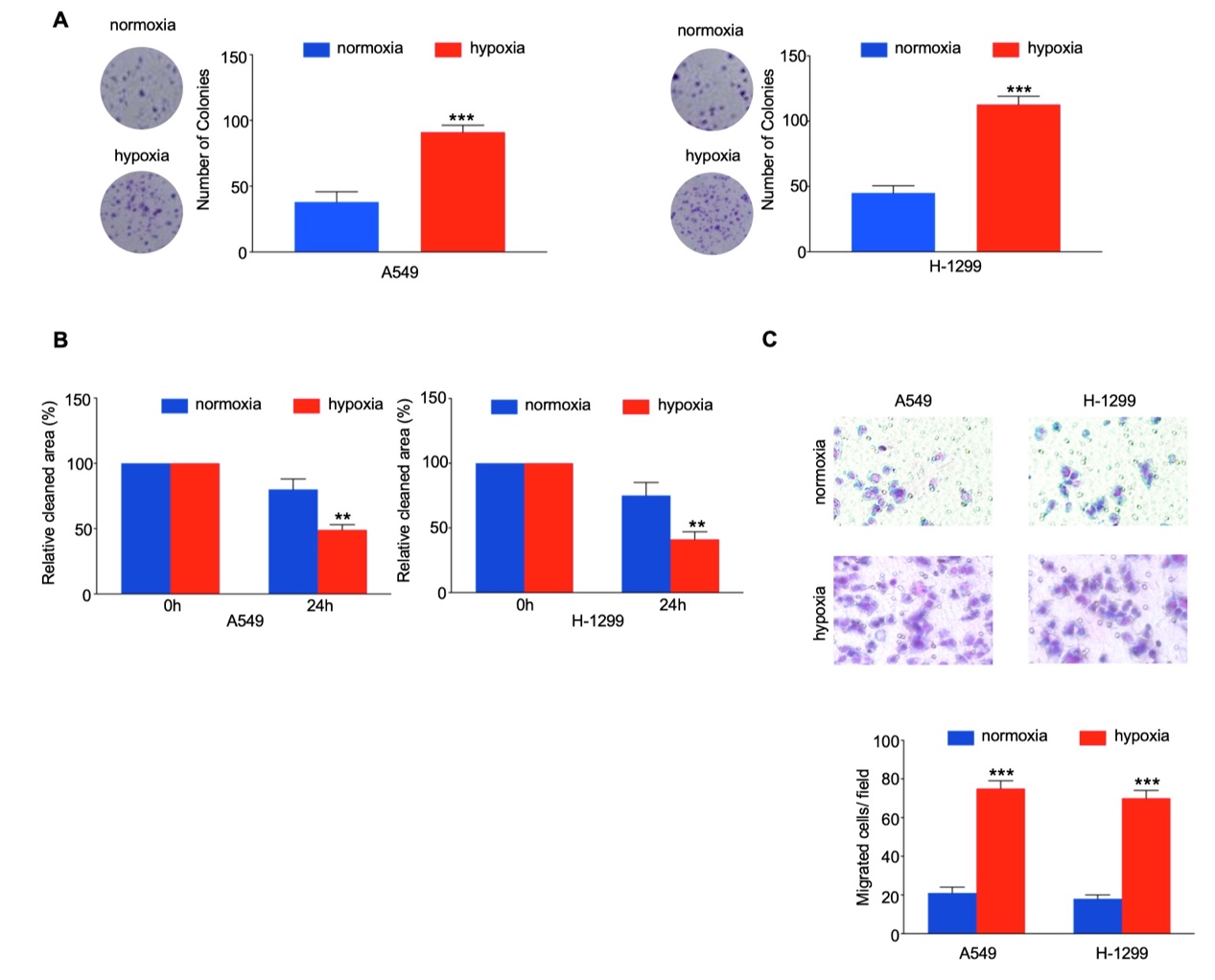
**

**FIGURE S4**

| Table-S1: Basic information of datasets included in this study for identifying hypoxia-related gene set | | | | | |
| --- | --- | --- | --- | --- | --- |
| **Accession number /Source** | **Platform** | **Number of  patients** | **Stage** | **Sex** | **Survival data** |
| GEO: GSE50081 | Affymetrix Human Genome U133  Plus 2.0 Array | 181 | I:127 II:54 | Female:83 Male:98 | OS |
| GEO: GSE30219 | Affymetrix Human Genome U133  Plus 2.0 Array | 289 | I/II:240 III/IV:49 | Female:42 Male:247 | OS |
| GEO: GSE37745 | Affymetrix Human Genome U133  Plus 2.0 Array | 196 | I/II:165 III/IV:31 | Female:89 Male:107 | OS |

**Figure S1** (**A**) Pathways involved in epithelial-mesenchymal transition (EMT), IL-6/JAK2/STAT3, PI3K/Akt/MTOR, Hypoxia, MYC targets V1 and Angiogenesis were activated in Cluster 2 (worse survival), whereas Cluster 1 (favorable survival) in the GSE39582 cohort by GSVA.

**Figure S2** (**A**) The correlation of 22 immune cells proportions obtained from 666 lung cancer patients in GEO databases; (**B**) and (**C**) LASSO regression was performed, calculating the fitting criteria.

**Figure S3** CXCL6 is the critical chemokine induced by hypoxic NSCLC cell to recruit TANs derived from peripheral blood. (A) Quantification of neutrophil migration as assessed by transwell assays; (B) and (C) Expression of CXCL6 in hypoxic or normoxic NSCLC cells was examined by real-time PCR and ELISA; (D) Quantification of neutrophil migration as assessed by transwell assays.

**Figure S4** TANs derived from peripheral blood promote NSCLC cells proliferation, migration and invasion. (**A**), (**B**) and (**C**): NSCLC cells cocultured with TANs or alone were subjected to colony formation, wound healing, and transwell invasion assays.
